# Video conferencing versus telephone calls for team work across hospitals: a qualitative study on simulated emergencies

**DOI:** 10.1186/1471-227X-9-22

**Published:** 2009-11-30

**Authors:** Stein R Bolle, Frank Larsen, Oddvar Hagen, Mads Gilbert

**Affiliations:** 1 University Hospital of North Norway, Norwegian Centre for Integrated Care and Telemedicine, 9038 Tromsø, Norway; 2 University Hospital of North Norway, Division of Trauma Care and Pre-Hospital Services, 9038 Tromsø, Norway

## Abstract

**Background:**

Teamwork is important for patient care and outcome in emergencies. In rural areas, efficient communication between rural hospitals and regional trauma centers optimise decisions and treatment of trauma patients. Little is known on potentials and effects of virtual team to team cooperation between rural and regional trauma teams.

**Methods:**

We adapted a video conferencing (VC) system to the work process between multidisciplinary teams responsible for trauma as well as medical emergencies between one rural and one regional (university) hospital. We studied how the teams cooperated during simulated critical scenarios, and compared VC with standard telephone communication. We used qualitative observations and interviews to evaluate results.

**Results:**

The team members found VC to be a useful tool during emergencies and for building "virtual emergency teams" across distant hospitals. Visual communication combined with visual patient information is superior to information gained during ordinary telephone calls, but VC may also cause interruptions in the local teamwork.

**Conclusion:**

VC can improve clinical cooperation and decision processes in virtual teams during critical patient care. Such team interaction requires thoughtful organisation, training, and new rules for communication.

## Background

Time-critical medical emergencies require rapid recognition of important clinical signs and symptoms in order to diagnose and stabilise vital functions while treating the patient. Efforts to improve treatment in these settings transcend individual deeds, and should focus on human factors, actions and interactions in teams [1]. Difficult emergencies may also require teams of specialists not available in rural hospitals. "Virtual teams" can be established during such situations, when team members use interactive communication technology combining picture and sound to stay in touch. Video conferencing (VC) used for medical emergencies may reduce the number of patients transferred to trauma centers [2-4] and offer a quality of clinical service not previously available [5-7]. This may reduce discrepancies between urban and rural trauma care [8].

Virtual teams may add complexity [9], disturb work flow and provoke lack of confidence in medical emergencies, hampering patients treatment. Thus understanding of human and organisational problems related to communication is needed to assess when accessory communication technologies are useful or harmful [10]. So far, use of VC in emergency medicine have expanded the local team with only one specialist via the video link, and most clinical studies refer to minor trauma and fairly simple patient conditions.

We studied if VC could improve communication and team function between rural and central emergency hospital teams with several participants at either side of the video link. Searching for evidence beyond measures and numbers [11], we chose a qualitative approach to find strengths and weaknesses of VC when compared to conventional telephone calls during simulated, complex trauma and emergency medicine cases.

## Methods

### Participants

We adapted a commercial off-the-shelf video conferencing technology to fit medical emergencies between a rural hospital and an university hospital in a remote arctic area of North-Norway. The rural partner was Longyearbyen Hospital (LYB), located on Spitsbergen, about 1.200 km north of the University Hospital of North Norway (UNN), Tromsø, Norway. The rural hospital has three emergency teams, all included in the study. The teams have three members, a doctor (GP or a surgeon), an operating room (OR) nurse and a nurse anesthetist. Each LYB team was paired with one of three trauma teams at UNN, each with specialists in surgery, neurosurgery, intensive care and emergency medicine. The specialists were appointed by the clinical head of each department based on available staff, and all accepted to participate in the study. The trauma teams were assisted by nurses and flight coordinators on duty in the Emergency Medical Dispatch center (EMD) situated in the Department of Emergency Medicine at UNN.

### Communication technology

The VC system has two-way video and audio. Two cameras were installed in the emergency room at LYB; one camera above the patient bed and one wall-mounted overview camera. Both cameras have pan, tilt and zoom. Physiological variables with trends (ECG, heart rate, blood pressure, oxygen blood saturation and temperature) can be viewed real time at both locations. At UNN, the VC system was installed with one camera and two 37¨ wall-mounted widescreens in the conference center of the EMD. The primary design concept was to minimise the amount of technology interaction for the team working around the patient in the rural emergency room. The technology therefore can be remotely controlled from the EMD. For data compression and decompression of video streams we used two Tandberg video codecs (Tandberg, Lysaker, Norway), connected with a 2 MB/s data network.

For comparison, we also tested virtual team building without the VC system, using conventional telephones for communication between hospitals. The available telephones were a mix of wall-plugged units, wireless handsets, and loud-speaking telephone conference units.

### Simulation trials

We tested the "virtual emergency team" in simulated emergency scenarios (Table 1). The patients were healthy volunteers, instructed to play symptoms and signs, and given realistic appearance by professional make-up. Physiological variables were generated by simulators and displayed real time on monitors, at both locations during VC, and at LYB only during telephone scenarios. A facilitator (SRB) provided additional information, such as respiratory sounds and urine color, when participants asked for it. Participants had no former experience in using the VC system. They were given a 15 minutes introduction on how to use it followed by a practical training session (case A, Table 1). The same team members at both hospitals cooperated on another two scenarios (case B - E, Table 1). Team 1 used videoconferencing for all scenarios while team 2 and 3 explored both communication modes. The teams were allowed to work 45 minutes on each case.

**Table 1:** The scenarios in brief as presented for the three teams.

	Team (communication mode)	Case descriptions
Case A	Team 1 (video), Team 2 (video), Team 3 (video)	Preparatory video training session: Female, 24 years, car accident, pelvic fracture. Hypovolemia, falling blood pressure, increasing heart rate.

Case B	Team 1 (video)	Male, 54 years, acute myocardial infarction and infectious condition, cardiac arrest, advanced CPR and intubation prior to hospital arrival. Unconscious (Glascow Coma Scale 6 - 7), body tp 40 C.

Case C	Team 1 (video)	Male, 30 years, burn accident with inhalation burn, 40% skin injury, CO-intoxication. Respiratory frequency 35, SpO2 99%, disoriented, some pain.

Case D	Team 2 (audio), Team 3 (video)	Male, 27 years, fall accident, head injury, pneumothorax. At hospital arrival intubated, Glascow Coma Scale 4, elevated intracranial pressure, BP 150/100 - 200/150, HR 60 - 45

Case E	Team 2 (video), Team 3 (audio)	Female, 25 years, snowmobile accident, head injury, initially unconscious. At hospital arrival awake, but rapidly deteriorating level of consciousness, bleeding wound left forehead, unilaterally dilated pupil, left temporal epidural hematoma.

### Data collection and analysis

An external observer (FL) followed each scenario, focusing on intra- and inter-group communication. Semi-structured group interviews were conducted following each of the nine simulated scenarios (case A - E, Table 1). Group interviews facilitate interaction and exchange of ideas between the informants, and was chosen to allow team members to discuss their experience, behaviour, group dynamics and how the team could work better together. Prepared open-ended questions were combined with questions based on observations during the play. Concepts like interaction, cooperation, media richness, social presence, awareness and implications for medical treatment were used to develop the interview guide.

The scenarios were video taped and the interviews recorded and transcribed. The transcribed material was coded with regard to the themes in the interview guide, and sections concerning changes of work related to the use of video communication were labeled. We analyzed this material using an abductive approach [12-14], a notion we apply to the process of moving from lay descriptions and meanings of social life to social scientific descriptions, concepts and theories. The concepts selected were conceptualization of communication and team work. The focus of our analysis was whether the participants acted differently because of the video communication. The interviews were analyzed and interpreted by an anesthesiologist (SRB) and a sociologist (FL), based on an understanding that technology enables and constrains social practices [15]. Video recordings of the scenarios were analyzed to confirm observations made during the scenarios and interpretations of the transcribed interviews. Quotes were chosen to illustrate main concepts discussed by participants.

## Results

### Observations

In each scenario, communication was initiated by LYB, with request for medical advice and transportation of patient. UNN doctors were contacted "on demand" and met in the EMD during both communication modes. Several phone calls were needed to solve telephone scenarios, during which the doctor at LYB usually left the patient room. When using VC, the doctor stayed bedside continuously, and the VC was kept active for the remaining time of the scenarios. The specialists made comments and suggestions based on their visual input. When able to see the patient, they suggested more active treatment. Due to technical limitations, the UNN team had to choose two out of three video sources on their local screens. At times they chose not to display vital signs, which caused misunderstandings within the group. Thus, important changes in clinical parameters were missed when both sites relied on the other.

### Interviews

The doctor at the remote hospital was considered the leader in charge of patient care regardless of communication technology. Traditionally, doctors at the remote hospital act as a link in the communication between the nurses at the primary hospital and university hospital. During VC, the nurses found it easier to address the specialists directly and vice versa. LYB teams were more comfortable when questions and messages from the specialists were given to all team members because questions from the nurses would not be transmitted through the local doctor [Appendix 1A].

UNN specialists wished to start communication earlier than those at LYB. Some wanted to be on-line before the patient arrived. LYB doctors, on their side, wanted to be prepared when talking to their colleagues, not wasting valuable resources at the university hospital, and suggested initiation of VC after initial examination and stabilisation of patients [Appendix 1B].

Participants said VC made it possible to work more effectively and as one team. However, cooperation between participants is different on VC than with telephones, and need to be learned [Appendix 1C]. Communication and team work had to be balanced with the need for working independently. Rules for communication had to be set. Most important, those at LYB wanted to work uninterrupted during the first minutes after patient arrival. UNN doctors noticed that discussions of diagnostics and treatment within their own group were disturbing the LYB teams, which at times made them mute their microphone. This option was agreed should be used more [Appendix 1D].

Team members at LYB said they relied on oral information, and did not need images of their colleagues at UNN. Still they found facial contact to ease communication, and VC beneficial for time-critical clinical decisions. Also, they believed visual information gives a better foundation for decision-making and had more confidence in treatment advice given by specialists during VC [Appendix 2A].

The specialists said VC made it possible to use "the clinical eye" and that they sometimes observed matters that LYB doctors or nurses did not. They said it was valuable to monitor the effects of treatment, for example improved blood pressure after intravenous fluid or the reaction on light of a dilated pupil following intensive treatment efforts. Some specialists felt more psychological involvement and commitment to the patient during VC, important when prioritising between patients [Appendix 2B].

All believed good patient care were given during both communication modes, but several thought VC improved quality. The use of telephones may result in rumors, while VC improve the certainty and safety, and ensure quality of treatment, doctors said. Doctors at LYB said they might be more willing to do certain procedures with direct guidance and visual support via VC, even given procedures they do not perform ordinarily. UNN specialists came together to discuss, which was believed to result in better decisions and improved treatment of patients regardless of communication technology [Appendix 3]. Prior to trials, most members at LYB expressed uneasiness about the possibility of being "surveilled". Following trials, they reported less stress than anticipated, and stated that stress decreased after each VC scenario. Nurses reported increased tension when the doctor left the patient room to make telephone calls, in particular when patient condition deteriorated, and argued that this made VC favorable [Appendix 4].

## Discussion

### Virtual team building

In this study, VC improved clinical work processes in virtual trauma teams when compared to telephone communication. VC increased interaction among team members providing both oral and visual information, and many-to-many communication. This improved possibilities for multi-tasking, which is important for the efficacy of trauma teams [16]. Improved information made team members more confident about advice they gave or received when using VC. Seeing the patient made specialists more involved in patient care, which may result in more active treatment [17].

Doctors in tertiary trauma centers are likely to be more used to early scramble of trauma teams than those at hospitals with low trauma frequency. This explains why the university hospital doctors were more willing than local doctors to accept over-triage through early initiation of virtual trauma teams. While rural hospital doctors wanted to prepare for VC in the same manner as for telephones, specialists found it useful to observe patients and treatment during some time when advising for further action. We suggest criteria-based initiation of virtual dual-site trauma teams, locally adapted based on available resources at both locations [18].

Complex medical problems increase the need for communication between colleagues, as do larger teams. Comprehension, interpretation, conflict resolution and communication are critical factors affecting the quality of the end result of teams in complex environments [19-21]. Novel technologies may add to this complexity [9]. Although not arguments against VC in itself, such issues can be more visible than during phone calls. Participants in this study were quickly able to cooperate effectively, and specialists may through their expertise simplify the complexity of medical problems. Still individuals and teams should be trained in communication and leadership [19,22,23], also when working in a virtual setting.

### Communication technology and adverse effects

Innovative communication technology used in a medical environment may enhance, but also interrupt, clinical work processes. In this study telephones were considered as discontinuous communication when compared to VC, while interruptions happened more easily during VC. The telephone has been used for many years and there are established rules, although informal, for the use of it. The use of social protocols and new technical solutions should be explored in order to decrease interruptions during VC.

Compression and decompression of video signals leads to latency which can be disruptive to clinically effective telepresence. This problem can be solved by using ultra broadband networks [5,17], but is not yet possible in many areas of the world for economical or technical reasons.

When VC was not used, rural hospital doctors had to make several phone calls to discuss deteriorating patient conditions and requesting patient transferal. In our setup, we found telephones required staff to have more attention on communication technology than during VC, with reduced attention on clinical work.

Participants asked whether loud-speaking telephone conference units (speakerphones) at both sites would be as useful as VC for many-to-many communication. VC is beneficial for following conversations with multiple participants [24]. Thus, audio-only speakerphones between multiple participants used in a time-critical setting, may cause more interruptions than VC for the rural hospital team. Also, specialists found discussions easier when observing, and the availability of video has been shown to dramatically influence the use of a team's conferencing system [25]. Therefore, it is reasonable to believe that speakerphones are less likely to be used, and would have several drawbacks when compared to VC. Specialists had to leave their own working environment for participation in VC. This is usually more interruptive to their work than phone calls from a remote hospital doctor. The added benefit of more information through VC, working in a team with other specialists contributing to the case, and the cooperation with the EMD for planning of patient transportation may outweigh this disadvantage. We found VC to cause misunderstandings, when vital signs were not displayed at both hospitals. Similarly, important information can be missed when microphones are muted, or cameras inadequately focused. These issues can be solved by forcing a different setup of computer screens, different user interfaces or by improved training.

VC has been used and studied in various settings for decades. Video has been shown to support interactions within teams, but important design issues need to be met or VC systems are not used [24-26]. The overhead of setting up and planning VC meetings should not be added to the tasks of a small rural hospital team in a time critical situation. In our setup this is the responsibility of the university hospital, whose team also remotely controls all cameras. Synergies between technology and work processes are important for successful implementations of technology in health care [27].

### Methodological issues

We did not tell participants to work in any particular way with either technology. This may have limited the use of technology to it's full potential. For example, doctors at LYB usually left the patient room to make telephone calls. This was mentioned as a drawback with telephones, but was due to tradition rather than technical limitations.

Participants received very little pre-training and had hardly any time to familiarize themselves with the VC system prior to this study. Compared with years of experience of telephone communication, the comparison between the use of the two technologies is less valid. However, not all potential team members in large hospitals can have extensive training in virtual emergency teams, and we believe our study reports typical experiences of new members in such teams.

This study may be biased because of the novelty factor associated with new technology, the unfamiliar setting [10], the Hawthorne effect [28], the use of simulations rather than real patients, and by researchers searching for effects of technology and extended teams. After concluding our study, several participants from both hospitals have requested VC in emergency situations, and the system is in clinical use. We believe this strengthens our main conclusions.

Qualitative research methods, as used in this study, are useful for the study of human experience, communication and processes, especially related to interaction and activity, but cannot be applied to answer questions about numerical matters such as extent and distribution [11]. The team to team cooperation between rural and regional trauma teams should be further investigated, and new quantitative studies may address issues discovered by our qualitative approach.

## Conclusion

VC can improve communication between hospital teams responsible for treating and triaging emergency patients, through images, vital signs, and increased interaction between team members at either side of the video link. Increased size of the consulted team may cause more interruptions to work flow around the patient when using VC, but the experts can be more involved in decision processes. VC increases likelihood of gaining a common understanding and support simultaneous work.

VC facilitates the availability of the university hospital's medical expertise and advise despite extremely long communication lines and challenging patient logistics. This cuts the time before patients are seen by specialists, and may positively affect outcome. Socio-technical design of clinical VC systems, minimising interruptions, training of virtual teams and adaptation of working routines are important issues when implementing future systems.

## Competing interests

The authors declare that they have no competing interests.

## Authors' contributions

SRB, and MG have contributed to conception and design of the study, acquisition and analysis of data and drafted the manuscript. FL has contributed to design of the study, acquisition and analysis of data and helped drafting the manuscript. OH has contributed to conception and design of the study, and acquisition of data. All authors read and approved the final manuscript.

## Appendix 1: Team work, work flow and communication. Excerpts from interviews

A: Responsibility and team communication

LYB-doctor: VC does not change my responsibility, I still have that.

LYB-nurse: If our doctor talk with an anesthesiologist down at UNN and pass that message to me, it is often a possibility for misunderstandings. It is a lot better when I can talk directly.

LYB-nurse: I ask directly, and immediately get the message I need. One saves time and frustration.

LYB-doctor: With telephones I become a connecting link in passing on information. We lose a lot on that.

LYB-doctor: With VC everyone got all information at once.

LYB-nurse: If everyone is updated all the time, we are stronger.

B: When to initiate video conferencing

LYB-doctor: As a doctor here we need to see the patient first. We can not call UNN at once. (...) UNN might not have too much personnel for establishing inter-hospital trauma-teams.

UNN-specialist: I think we should take part from the beginning. (...) It is very important for us to get the same report as LYB when the patient arrives. It is invaluable.

UNN-specialist: In this scenario, I felt the patient was presented to us too late. It would be better if we could watch when the patient arrived.

C: Team work

LYB-doctor: As if they were somewhere in the room, as if they talked across the table.

LYB-nurse: I think we can work quicker and more effectively in this way.

LYB-doctor: They are also a part of the team, because when they have been with the patient for a while, they will also follow the parameters just like us and see development.

LYB-nurse: We only need to learn how to work during VC, then I don't think there are drawbacks at all.

D: Interruptive communication

UNN-specialist: We agreed with them that we should mute our microphone while they did examinations.

UNN-specialist: I think it is very important that we take part from the very beginning, but that we keep silent and not interrupt before the initial work has been done.

LYB-nurse: They (UNN-specialists) need to learn to watch without talking.

UNN-specialist: It was almost like being there. And that makes us maybe too eager. (...) We should have muted our microphone more often.

LYB-doctor: I believe in a quite, uninterrupted, initial examination of the patient.

**UNN** = University Hospital of North Norway. **LYB** = Longyearbyen Rural Hospital. **VC** = Video Conferencing.

## Appendix 2: Importance of visual input. Excerpts from interviews

A: Observation of teams and team work

LYB-nurse: I don't think we need the image from UNN (...) it is for them it should be of value, and then we benefit from it.

LYB-nurse: It is the direct communication that is important, just like a loudspeaker (...) but then we would have to describe things in much more detail.

LYB-doctor: I think the quality during VC is better, because they are more involved in what we do.

UNN-specialist: I believe we get more useful information with VC. (...) to see what they do (...) and how.

LYB-doctor: (With telephones,) sharing information becomes worse, that is almost obvious. One person has to communicate everything. There are limitations with that, and specialists don't get the total overview as they do when they see and observe themselves.

UNN-specialist: It is about complexity. If it is simple and easy to get an overview, I think telephone is just as good. If it is complex and critical and the order of your decisions matters, then decisions made when seeing would absolutely be different.

B: Observation of patient and vital signs

UNN-specialist: The combination of seeing vital data, following it live, feeling that you take part in development, taking part in time and place, it means a lot. (...) You get a more complete overview, which I believe affects decisions.

UNN-specialist: To see the pupils of a patient is of great value to me. Often times we get strange information from a submitting doctor. We have experienced many times that the pupils are described as reacting to light, and they are not.

UNN-specialist: You may observe other matters than those at LYB, and get a different understanding.

UNN-specialist: There is less interpretation.

UNN-specialist: I was more passive during telephone conferencing.

UNN-specialist: To get a piece of the patient, and to talk with the others and see their faces, it matters, really.

LYB-doctor: I find them a lot more involved in the patient. (...) Now they are here.

**UNN** = University Hospital of North Norway. **LYB** = Longyearbyen Rural Hospital. **VC** = Video Conferencing.

## Appendix 3: Implications for medical treatment. Excerpts from interviews

LYB-doctor: The work flow and the contact with UNN, the support, everything was better during VC.

LYB-nurse: Of course you definitely avoid sources of error when you get information directly.

UNN-specialist: Often times with telephones there are a lot of rumors. Different doctors are informed by different people, and they start talking, and the OR program is stopped. Then a cascade of things occurs. When the patient arrives a couple of hours later the story is totally different. It is therefore very good to observe what takes place.

LYB-doctor: I am perhaps more worried when they did not see what we did, and maybe they did not get all the information either. They didn't ask have you done this, have you done that.

LYB-doctor: It feels a lot more safe that somebody sits there and take part in the decision process and supervise the patient, see the patient.

LYB-nurse: I could ask at once. But if we did not have him (the anesthesiologist) here, if I had to use a telephone, I would first have to start infusion of sedation and asked afterwards. Otherwise the patient could have woken up.

LYB-nurse: Of course, the better and more correct impression they (UNN-specialists) have in the situation, the better help they can give. (...) They have better premises to give advice.

LYB-doctor: I could have done trepanation, but only on vital indication. (...) It could be good guidance with VC. I don't think I would do that without VC.

UNN-specialist: Cooperation between specialists is quicker when we sit together. And it is much easier to discuss when we observe.

**UNN** = University Hospital of North Norway. **LYB** = Longyearbyen Rural Hospital. **VC **= Video Conferencing.

## Appendix 4: Stress and confidence. Excerpts from interviews

LYB-nurse: I felt it very positive that they were here. I was very sceptical in the beginning.

LYB-nurse: I was more confident in the VC situation. That's why I like it.

LYB-nurse: When our doctor was outside the patient room and the patient conditioned worsened, did not reply to verbal contact, then I disliked that he was not here.

LYB-doctor: VC gives me more confidence.

**UNN** = University Hospital of North Norway. **LYB** = Longyearbyen Rural Hospital. **VC** = Video Conferencing.

## Pre-publication history

The pre-publication history for this paper can be accessed here:

http://www.biomedcentral.com/1471-227X/9/22/prepub
